# The Role of Self-Control and Early Adolescents’ Friendships in the Development of Externalizing Behavior: The SNARE Study

**DOI:** 10.1007/s10964-015-0287-z

**Published:** 2015-04-29

**Authors:** Aart Franken, Terrie E. Moffitt, Christian E. G. Steglich, Jan Kornelis Dijkstra, Zeena Harakeh, Wilma A. M. Vollebergh

**Affiliations:** 1 Department of Interdisciplinary Social Science, Utrecht Centre of Child and Adolescent Studies, Utrecht University, PO Box 80.140, 3508 TC Utrecht, The Netherlands; 2 Duke University, Durham, NC USA; 3 King’s College London, London, UK; 4 Department of Sociology, Interuniversity Center for Social Science Theory and Methodology, University of Groningen, Groningen, The Netherlands

**Keywords:** Alcohol use, Antisocial behavior, Self-control, Social network analysis, SIENA, Tobacco use

## Abstract

This social network study investigated the moderating role of self-control in the association between friendship and the development of externalizing behavior: Antisocial behavior, alcohol use, tobacco use. Previous studies have shown inconsistent findings, and did not control for possible friendship network or selection effects. We tested two complementary hypotheses: (1) That early-adolescents with low self-control develop externalizing behavior regardless of their friends’ behavior, or (2) as a result of being influenced by their friends’ externalizing behavior to a greater extent. Hypotheses were investigated using data from the SNARE (Social Network Analysis of Risk behavior in Early adolescence) study (*N* = 1144, 50 % boys, *M*_age_ 12.7, *SD* = 0.47). We controlled for selection effects and the network structure, using a data-analysis package called SIENA. The main findings indicate that personal low self-control and friends’ externalizing behaviors both predict early adolescents’ increasing externalizing behaviors, but they do so independently. Therefore, interventions should focus on all early adolescents’ with a lower self-control, rather than focus on those adolescents with a lower self-control who also have friends who engage in externalizing behavior.

## Introduction

Early adolescents’ development of externalizing behaviors is influenced by their friends’ externalizing behavior (see Veenstra et al. 2013). Such externalizing behaviors include antisocial behavior, alcohol use, and tobacco use. Early adolescents’ self-control is also associated with the development of such externalizing behaviors (e.g., de Kemp et al. 2009). Several studies have investigated a potential moderating effect of self-control on the tendency to adapt friends’ externalizing behavior, focusing primarily on the outcome of delinquency. These studies have provided inconsistent findings: Past research has suggested that higher self-control might be associated with a lower likelihood to adapt behavior based on delinquent peers (Gardner et al. 2008; Wright et al. 2001), that self-control might not moderate this association (McGloin and O’Neill Shermer 2009), or that high self-control might even be associated with a higher likelihood to adapt such behaviors (Meldrum et al. 2009).

The inconsistency in the above findings might stem from several different sources. Some of the studies used adolescents’ reports of their own and of their peers’ behavior, which is likely to be biased. Further, these studies did not investigate the continuous co-development of externalizing behavior and friendship over time, did not take friendship selection effects into account, nor did they take both friends’ delinquency and the network structure (how friendships are embedded in the network of peers) into account (see Veenstra et al. 2013). The present study aimed to overcome these limitations by using a data set of adolescents’ social networks measured repeatedly over time, and by modeling the co-evolution of the network (friendship) and the behavior (externalizing behavior), using stochastic actor-based modeling (SABM; Steglich et al. 2010). SABM modeling allows disentangling selection effects (adolescents become friends with those who have the same characteristics) from influence effects (friends become more similar to each other over time), and takes the friendship network structure into account. Furthermore, although previous studies focused on delinquency, we investigated a composite of multiple externalizing behaviors (antisocial behavior, alcohol use, and tobacco use). This allowed for a more complete understanding of the role of self-control during early adolescence. In sum, this study aimed to test the role of self-control in the spread of externalizing behavior during early adolescence; by studying how self-control is associated with adapting friends’ externalizing behavior hereby using longitudinal social network analyses.

### Adolescent Onset of Externalizing Behavior

It was important for our study to capture the window of entry into early adolescence, because it is the peak developmental period for initiation of externalizing behaviors. Early adolescents become increasingly engaged in externalizing behaviors such as antisocial behavior, alcohol use, and tobacco use (e.g., Currie et al. 2012; Jennings and Reingle 2012). This sudden increase of externalizing behavior has been explained by the dual-taxonomy model (Moffitt 1993). According to this model, adolescents are motivated to overcome the stressful experience of the “maturity gap”. This gap is experienced when adolescents feel biologically mature, but do not yet receive adult-like rights and privileges from society. Mimicking externalizing behavior of peers is a way for these adolescents to obtain an adult-like status among their peers, thus bridging the maturity gap. Few studies have tested whether bridging the maturity gap through externalizing behavior depends on the adolescents’ pre-existing personality characteristics, such as self-control.

Recent social network studies, using SABM, have been able to disentangle selection effects (adolescents select friends who are similar to them) from influence effects (adolescents become more similar to their friends), while taking the structure of friendship networks into account. These studies have shown that adolescents do mimic their friends’ externalizing behavior. Early adolescents not only select friends who are similar to them in antisocial behavior, alcohol use, or tobacco use, but they also adapt their behavior to become more similar to their friends (Burk et al. 2012; Huisman and Bruggeman 2012; Kerr et al. 2012; Light et al. 2013; Mercken et al. 2012; Osgood et al. 2013; Steglich et al. 2012). However, some studies found inconsistent or non-significant effects (Knecht et al. 2010, 2011; Mercken et al. 2009, 2010a, b, 2012; Weerman 2011), indicating that other variables might be needed to better explain the co-development of friendship and externalizing behavior. As not all adolescents are equally susceptible to the influence of their peers (Brechwald and Prinstein 2011), it is important to investigate variables such as pre-existing personality—in particular personal self-control, which might moderate the likelihood to adapt friends’ externalizing behavior.

### Self-Control and Externalizing Behavior

Since the General Theory of Crime (Gottfredson and Hirschi 1990), the personality characteristic called self-control (i.e., self-regulation; inhibitory control) has been studied to explain engagement in antisocial behavior and substance use. According to this theory, self-control is important in explaining both delinquency and friendship selection. The main reason adolescents with a low self-control are likely to end up together is that they may not be attractive friends to others. That is, they can be “unreliable, untrustworthy, selfish, and thoughtless. They may however be fun to be with, they are certainly more risk-taking, adventuresome, and reckless than their counterparts” (Gottfredson and Hirschi 1990, p. 157). Therefore, those adolescents with low self-control who are adventuresome and have trouble making other friends are likely to end up together. At the same time, adolescents who have lower self-control are more likely to engage in delinquent acts. Thus, the association between delinquency and delinquent friends might be explained by self-control. Rather than selecting friends with a similar delinquent behavior, adolescents might select friends who have a similar self-control level and also engage in delinquent behavior.

In general, studies find that self-control plays an important role in the development of adolescent externalizing behavior. Impaired childhood self-control is highly important as it is associated with an abundance of negative life experiences, such as substance use, criminal offending, school dropout, or unplanned teenage pregnancies, and with negative long term health and financial outcomes (Moffitt et al. 2011). Furthermore, having lower self-control might impact adolescents’ susceptibility to externalizing behavior (Gardner et al. 2008; McGloin and O’Neill Shermer 2009; Meldrum et al. 2009). Lower self-control has been associated with engagement in externalizing behaviors, such as antisocial behavior (criminal offending, delinquency; Cauffman et al. 2005; Chapple 2005; De Kemp et al. 2009), and substance use (Larsen et al. 2010; Marschall-Lévesque et al. 2013). Furthermore, adolescents with lower self-control are more likely to have deviant friends (Evans et al. 1997; McGloin and O’Neill Shermer 2009).

On the one hand, in line with the General Theory of Crime (Gottfredson and Hirschi 1990), self-control might explain any increase in externalizing behavior independent of friends’ externalizing behavior. The influence of friends who engage in externalizing behavior might even decrease with lower self-control (Meldrum et al. 2009). If adolescents with low self-control are more likely to engage in externalizing behavior regardless of their friendships, there would be fewer potential for friends to further influence their externalizing behavior. On the other hand, following the basic associations of self-control with externalizing behavior and self-control with deviant friends, a moderating role of self-control has also been proposed. In line with the social amplification effect (Wright et al. 2001), lower self-control has been found to increase the influence of deviant peers (Gardner et al. 2008; Wright et al. 2001). Thus, adolescents with lower self-control might be more likely to be influenced by their friends who engage in externalizing behavior. In order to properly test these complementary hypotheses, friendship selection and influence processes should be investigated simultaneously and longitudinally. While investigating these hypotheses, it is important to take possible selection effects of self-control into consideration. Although Gottfredson and Hirschi (1990) expected adolescents to select their friends on self-control, Young (2011) found negligible selection effects based on self-control. When taking SES, sex, and grade level friendship selection effects into account, while controlling for triad closure, Young (2011) concluded that self-control is not important in the formation of friendships at school.

## Current Study

The current study investigated the moderating role of self-control in the co-development of friendship and externalizing behavior (i.e., antisocial behavior, alcohol use, and tobacco use) during early adolescence. Whole grade friendship networks at the start of secondary school in the Netherlands (when participants are around 12 years old) were investigated using data from the Social Network Analysis of Risk behavior in Early adolescence (SNARE) study. Furthermore, we take possible selection effects and the network structure into account, using SABM in a data-analysis package called SIENA. Our study tested the hypothesis that self-control moderates the likelihood of adapting friends’ externalizing behavior. Specifically, we tested two complementary hypotheses. First, we tested if lower self-control regardless of friends’ externalizing behavior would increase the development of externalizing behavior. Second, we tested if adolescents with lower self-control would be more likely to be influenced by the externalizing behavior of their friends. Additionally, we took friendship selection based on self-control into consideration. Based on recent findings (Young 2011), we did not expect any selection effects based on self-control. Studying the role of self-control in this association is important as self-control has been proposed to be an important characteristic to train to help prevent engagement in externalizing behavior during adolescence (Moffitt and Caspi 2001; Piquero et al. 2010).

## Methods

### Procedure and Participants

Participants included 1144 students (50 % boys), aged 11.1–15.6 (*Mean* 12.7, *SD* = 0.47), 97 % were born in the Netherlands (as were 87 % of their fathers and 88 % of their mothers). Of the participants, 46.1 % followed lower level education (including preparatory secondary school for technical and vocational training) and 53.9 % followed higher level education (including preparatory secondary school for higher professional education and university).

Hypotheses were examined using data from the SNARE study. This is an ongoing prospective cohort study that focuses on the interplay between social networks and the development of externalizing behavior. The participants were recruited from two high schools, one in the middle and one in the north of the Netherlands; ethical approval for the study was granted by the first author’s university (see also Dijkstra et al. 2015). From these schools, all first and second year students were approached to participate in Year 1; these students are referred to as the first cohort of participants. The next year a second cohort of students entered the first year of the schools, and also was approached to take part in the study; these latter students are referred to as the second cohort of participants. All eligible students and their parents received an information letter about the research, in which they were asked to participate. Students or their parents were asked to send a reply card or email within 2 weeks, if they wished to refrain from participation. In total, 1783 students participated in the SNARE study, and 28 students (2 %) refused to participate. For the present study, we only included the first-year students from the first and the second cohort, as we were interested in the early engagement in externalizing behavior during early adolescence.

We used data from the pre-assessment and the first three waves for this study. The pre-assessment was during the first weeks of secondary school (September). The first assessment took place in October (Time 1), the second in December (Time 2), and the third in April (Time 3) of the same academic year. During these assessments, a teacher and one or more research assistants were present. The research assistant gave a brief introduction and explained that participants’ answers would remain confidential and anonymous. During the assessment, students filled in a questionnaire on the computer during one classroom period, around 45 min. After the pre-assessment, this questionnaire contained, next to self reports, peer nominations using CS socio software (www.sociometric-study.com). Peer reported variables were assessed by asking participants questions about their classmates. Participants were presented with all names of their classmates on their computer screen in alphabetical order, starting with a random name. For some peer nomination questions it was optional to select peers outside the classroom (but within the SNARE sample), using a search function. Unlimited, both same and cross sex, nominations were allowed. The students who were absent at the day of assessment were, if possible, assessed within a month.

### Measures

#### Self-Reported Externalizing Behaviors (Time 1–Time 3)

At all three time points, participants reported their engagement in three forms of externalizing behavior, including antisocial behavior, alcohol use, and tobacco use. *Antisocial behavior* was measured by asking participants how often (using a five point scale, ranging between 0 and 12 or more times) they had been involved in 17 types of antisocial behavior during the last month; including stealing, vandalism, burglary, violence, weapon carrying, threatening to use a weapon, truancy, contact with the police, and fare evasion in public transport. For example, participants were asked to indicate “During the last month, how often did you…”, “steal something from a shop”, “skip school while you should have been in class”, or “get in touch with the police for doing something you should not do”. The scale was based on the 12 questions used frequently in Dutch research (Nijhof et al. 2010), and five additional items which reflect other important antisocial behaviors: weapon carrying, threatening to use a weapon, truancy, contact with the police, and fare evasion in public transport (e.g., Van der Laan et al. 2010). For *alcohol use*, participants used a 13 point scale (ranging from 0 to over 40 times) to report on how many occasions they consumed alcohol in the last month (Wallace et al. 2002). For *tobacco use*, participants used a seven-point scale (ranging from never to more than 20) to indicate how many cigarettes they smoked daily over the past month (e.g. Monshouwer et al. 2011). At Time 1, the average score on antisocial behavior was 0.05 (*SD* = 0.22), the average alcohol use was 0.24 (*SD* = 1.02), and the average tobacco use was 0.12 (*SD* = 0.70). Because data using continuous measures of externalizing behavior frequency were highly skewed, all externalizing behavior data were recoded as binary, indicating no engagement at all (0) or any engagement (1) in any of the three behaviors: antisocial behavior, alcohol use, and tobacco use. This recoding allowed for an examination of externalizing behavior engagement rather than the frequency of externalizing behavior engagement. An exploratory factor analysis (using maximum likelihood estimations and oblique rotation) tested if the externalizing behaviors loaded on a single factor; they loaded on one factor, explaining 55.3 % of the variance. Therefore, a composite variable, representing the number of different externalizing behaviors participants engaged in (i.e., antisocial behavior, alcohol, or tobacco use), was computed; resulting in scores between zero (no externalizing behaviors) and three (all externalizing behaviors).

#### Self-Control (Pre-assessment)

Self-control was assessed with a shorter 11-item Dutch version (Finkenauer et al. 2005) of the self-control scale (Tangney et al. 2004). This scale assessed the ability of the person to control him or herself; for example “I have a hard time breaking bad habits”, “I have trouble concentrating”, “I get carried away by my feelings” (Tangney et al. 2004). Participants could respond on a scale from (1) not at all, to (5) very much. To facilitate interpretation, these scores were recoded, with higher scores indicating higher self-control. For our analyses, the mean scores were used. Cronbach’s alpha was .77.

#### Friendship Nominations (Time 1–Time 3)

Participants were asked to name their best friends. Participants could nominate friends within their class and, afterwards, friends from their grade. Grade networks were used for the current analyses.

### Analysis Strategy

Descriptive statistics for each of the four social networks (i.e., 2 cohorts in 2 schools) were calculated, including the average age, percentage of boys, average externalizing behavior level, frequency of externalizing behavior per assessment, self-control level, and the missing fraction (i.e., absent participants) of the networks. Furthermore, the Jaccard index, showing the relative stability over time, was calculated.

All network analyses were conducted using SIENA (Simulation Investigation for Empirical Network Analyses), version 4 (278), in R. SIENA is actor based, and models the longitudinal co-evolution of social networks and individual characteristics (Ripley et al. 2014). SIENA estimates the changes in networks and behavior over time. While controlling for *structural network effects* (i.e., the structure of friendships in the network), SIENA estimates both *network dynamics* and *behavior dynamics* longitudinally. The changes in individual behavior were modeled as an increase or decrease in the number of externalizing behaviors participants engaged in (ranging from zero to three externalizing behaviors). SIENA estimates changes between two points in time. For the current analyses the dependent variables are the network ties (friendships) and the number of externalizing behaviors participants engaged in (antisocial behavior, alcohol use, and tobacco use). For these analyses, SIENA disentangles selection (network dynamics) from influence (behavior dynamics) processes. The outcomes of SIENA analyses are based on an iterative Markov chain Monte Carlo approach (Snijders et al. 2010; Ripley et al. 2014).

Commonly used *structural network effects* were added, and as suggested by the SIENA manual (Ripley et al. 2014, see also Veenstra et al. 2013) other network effects were added to optimally capture the friendship structure in the current networks. The effects which are generally included in SIENA analyses were network density, reciprocity, transitive triplets (likelihood to befriend friends of friends), three-cycles (indicates hierarchies), indegree popularity (square root version; likelihood for participants who receive many friendship nominations to receive extra friendship nominations), indegree activity (square root version; likelihood for participants who receive many friendship nominations to send extra friendship nominations), and outdegree activity (square root version; likelihood for participants who send out many friendship nominations to send out extra friendship nominations); for more details see Ripley et al. (2014). To improve model fit, density and indegree popularity were allowed to vary between assessment periods. Furthermore, transitive reciprocated triplets were modeled to estimate the likelihood for triads (a group of three friends) to reciprocate friendships.

Before examining study hypotheses, several factors potentially affecting the social network (i.e., *network dynamiceffects*) were estimated as covariates (see Veenstra et al. 2013). The effects of same-gender friendship selection (i.e., girls nominate girls; boys nominate boys; girls were coded as 0, boys as 1) were estimated as well as the effects of proximity by using adolescents’ classroom and school locations as covariates (School 1 consisted of four locations). The effects of gender on sending (ego) and receiving of (alter) friendship nominations also was controlled. To investigate possible selection effects, the likelihood of sending (ego) or receiving (alter) friendship nominations, and selecting similar friends, was modeled based on externalizing behavior and self-control. To test if lower self control would be associated with an increased likelihood of selecting friends who engage in externalizing behavior two interaction effects were added. These effects examine the potentially moderating role of self-control on friendship selection based on similarity in externalizing behavior. The first interaction effect (self-control ego × externalizing behavior similarity) models if self-control affects the likelihood for participants to select friends with a similar level of externalizing behavior. The second interaction effect (self control alter × externalizing behavior similarity) models if participants take their peers self-control into account when selecting friends based on similarity in externalizing behavior.

To test our main hypotheses, several *behavior dynamiceffects* (including influence effects) were estimated (see Veenstra et al. 2013). Behavior dynamic effects model changes in externalizing behavior. They model the rate of change, and whether behavior change conforms to linear or quadratic trends. A main effect of influence is estimated as the likelihood that participants adapt their externalizing behavior to become more similar to the average externalizing behavior of their friends (the “average alter effect”). A main effect of self-control was also modeled, testing our first hypothesis and estimating if self-control influences the likelihood for participants to change their externalizing behavior regardless of their friends’ externalizing behavior. Furthermore, to test our second hypothesis, an interaction effect between self-control and externalizing behavior was estimated. This effect modeled if participant’s self-control changes their likelihood to adapt their friends’ externalizing behavior.

## Results

### Descriptive Statistics of the Networks, and Externalizing Behaviors within Networks

Table 1 lists descriptive statistics for each of the four networks examined in this study. Results at Time 1 suggested that all four networks did not differ in age or antisocial behavior. There were some small differences in gender distribution, alcohol use, tobacco use, overall externalizing behavior, and self-control. None of the students of the smallest network, cohort 2 of School 2 used tobacco at Time 1.

**Table 1:** Descriptive statistics of friendship networks for School 1 (cohort 1 N = 432, cohort 2 N = 390) and School 2 (cohort 1 N = 186, cohort 2 N = 136), Time 1–Time 3

Variable	School 1		School 2	
	Cohort 1	Cohort 2	Cohort 1	Cohort 2
Age				
Time 1	12.65 (0.43)	12.65 (0.43)	12.66 (0.48)	12.70 (0.68)
% Boys				
Time 1*	0.50 (0.50)^a^	0.48^ab^ (0.50)	0.47^ab^ (0.50)	0.61^b^ (0.49)
Antisocial behavior				
Time 1	0.22 (0.41)	0.27 (0.45)	0.21 (0.41)	0.29 (0.46)
Time 2	0.25 (0.43)	0.27 (0.45)	0.21 (0.41)	0.29 (0.45)
Time 3	0.25 (0.43)	0.27 (0.45)	0.27 (0.44)	0.29 (0.46)
Alcohol				
Time 1*	0.11^ab^ (0.31)	0.14^a^ (0.34)	0.07^b^ (0.25)	0.05^b^ (0.22)
Time 2	0.11 (0.31)	0.10 (0.30)	0.07 (0.26)	0.09 (0.29)
Time 3	0.12 (0.33)	0.14 (0.35)	0.11 (0.31)	0.14 (0.35)
Smoking				
Time 1*	0.06^ab^ (0.31)	0.10^a^ (0.42)	0.01^b^ (0.11)	0.00^b^ (0.00)
Time 2	0.05 (0.28)	0.10 (0.42)	0.04 (0.20)	0.05 (0.22)
Time 3*	0.13^ab^ (0.44)	0.19^a^ (0.56)	0.05^ab^ (0.22)	0.09^b^ (0.29)
Externalizing behaviors				
Time 1				
1 behavior	17.82 %	19.23 %	15.05 %	24.26 %
2 behaviors	5.79 %	5.39 %	5.37 %	4.12 %
3 behaviors	2.08 %	4.87 %	0.05 %	0.00 %
Time 2				
1 behavior	22.45 %	17.95 %	15.05 %	22.06 %
2 behaviors	5.09 %	6.41 %	3.76 %	6.62 %
3 behaviors	1.85 %	2.82 %	2.15 %	1.47 %
Time 3				
1 behavior	19.91 %	21.78 %	20.43 %	22.06 %
2 behaviors	4.40 %	7.69 %	6.99 %	5.88 %
3 behaviors	3.94 %	3.85 %	1.61 %	2.94 %
Self-control				
Pre-assessment*	3.63^ab^ (0.87)	3.57^a^ (0.53)	3.75^b^ (0.61)	3.70^ab^ (0.67)
Missing fraction				
Time 1	0.01	0.03	0.05	0.01
Time 2	0.01	0.04	0.03	0.01
Time 3	0.03	0.03	0.02	0.02
Jaccard index				
Time1–Time 2	0.46	0.47	0.44	0.45
Time 2–Time 3	0.46	0.48	0.44	0.45

* One-way ANOVA between group differences at *p* < .05. Within each time point (i.e. row), *Mean* scores with different superscripts differ significantly from each other at *p* < .05; calculated with a post hoc Tukey honestly significant difference test

Table 1 also includes network characteristics for each cohort. There were between 1 and 5 % absent participants during the assessments. The Jaccard index indicates the relative stability of each network over time. The Jaccard indices were between .44 and .48, well within the desired range for longitudinal social network analyses (Veenstra et al. 2013).

### SIENA Estimates of Friends’ Influence and Self-Control

The outcomes of the SIENA analyses are shown in Table 2. First, the structural network effects model the network structure, and optimize the goodness of fit of the networks. For three out of four networks there was a negative density effect, indicating that participants are likely to be selective in their friendship nominations (they nominate less than half the network as friends). There was a positive reciprocity effect in all networks, indicating that participants are likely to reciprocate friendship nominations. There was a positive transitive triplet effect in all networks, which shows that participants are likely to be friends with the friends of their friends. Moreover, in School 1 there was a negative three-cycle effect. In combination with the positive transitive triplet effect, this indicated that there was hierarchy in the networks (within triads few participants receive many nominations, while many participants receive fewer nominations). Furthermore, as shown by a negative transitive reciprocated triplet effect in all networks, triads were less likely to have reciprocated ties than dyads, which is another indication of hierarchy in the network. Particularly in period 1 we found a positive indegree—popularity effect in two networks and a negative effect in another. This indicated that those with many friends were more likely to increase their number of friends (positive effect), or that they are less likely to increase their number of friends (negative effect). The negative effects of indegree—activity in all networks indicate that those participants who received many friendship nominations were less likely to send out nominations themselves. Last, the outdegree—activity modeled individual differences in the number of friends nominated by participants, this was positive in three networks indicating that those with a higher outdegree were more likely to increase the number of friends they select.

**Table 2:** Estimates of effects for externalizing behavior, self-control, and the friendship networks for two schools, and two cohorts for Time 1–Time 3

Variable	School 1		School 2	
	Cohort 1	Cohort 2	Cohort 1	Cohort 2
Network dynamics				
Outdegree (density)				
Period 1	−2.23* (0.24)	−2.46* (0.18)	−2.51* (0.33)	0.57 (1.22)
Period 2	0.06 (0.19)	0.25 (0.20)	0.09 (0.26)	−0.44 (0.49)
Reciprocity	2.86* (0.08)	2.38* (0.08)	2.36* (0.18)	2.65* (0.21)
Transitive triplets	0.55* (0.02)	0.48* (0.02)	0.48* (0.04)	0.58* (0.06)
Transitive reciprocated triplets	−0.51* (0.03)	−0.37* (0.02)	−0.44* (0.06)	−0.37* (0.07)
3-cycles	−0.05* (0.02)	−0.10* (0.02)	−0.03 (0.04)	−0.06 (0.07)
Indegree—popularity (sqrt)				
Period 1	0.08* (0.03)	−0.08* (0.03)	0.20* (0.04)	−0.01 (0.07)
Period 2	−0.10 (0.06)	−0.21* (0.07)	−0.05 (0.08)	−0.22 (0.15)
Indegree—activity (sqrt)	−1.04* (0.10)	−0.73* (0.08)	−1.13* (0.25)	−1.81* (0.55)
Outdegree—activity (sqrt)	0.14* (0.02)	0.15* (0.03)	0.29* (0.06)	−0.01 (0.08)
Sex alter	−0.18* (0.04)	−0.06* (0.04)	0.10 (0.07)	−0.21* (0.08)
Sex ego	0.02 (0.05)	0.05 (0.05)	−0.15 (0.09)	−0.61* (0.21)
Sex similarity	0.68* (0.04)	0.84* (0.05)	0.66* (0.07)	0.58* (0.10)
Class similarity	0.42* (0.04)	0.34* (0.04)	–	–
Location similarity	0.70* (0.05)	0.82* (0.05)	0.92* (0.07)	0.54* (0.10)
Self-control alter	0.02 (0.03)	−0.01 (0.03)	−0.09* (0.04)	−0.08 (0.05)
Self-control ego	−0.10* (0.03)	0.01 (0.04)	−0.05 (0.06)	0.05 (0.08)
Self-control similarity	0.32* (0.11)	0.09 (0.13)	0.93* (0.20)	−0.37 (0.28)
Externalizing behavior alter	0.04 (0.04)	0.19* (0.04)	0.05 (0.09)	−0.02 (0.12)
Externalizing behavior ego	0.09 (0.05)	0.30* (0.04)	0.11 (0.10)	0.67* (0.23)
Externalizing behavior similarity	0.45* (0.19)	0.99* (0.13)	0.86* (0.37)	−0.10 (0.48)
Self-control ego × externalizing behavior similarity	0.04 (0.18)	−0.34 (0.18)	0.31 (0.36)	−0.43 (0.40)
Self-control alter × externalizing behavior similarity	0.01 (0.17)	0.15 (0.18)	0.20 (0.34)	−0.72 (0.39)
Behavior dynamics				
Externalizing behavior change period 1	1.33* (0.23)	1.48* (0.22)	1.41* (0.29)	1.65* (0.43)
Externalizing behavior change period 2	1.56* (0.23)	1.89* (0.31)	1.58* (0.35)	1.15* (0.29)
Externalizing behavior change linear shape	−1.26* (0.11)	−1.22* (0.12)	−1.44* (0.20)	−1.17* (0.20)
Externalizing behavior change quadratic shape	0.25* (0.08)	0.13 (0.09)	0.33* (0.14)	0.20 (0.15)
Externalizing behavior influence	1.15* (0.33)	1.17* (0.27)	0.93 (0.60)	1.24* (0.57)
Effect from self-control	−0.16 (0.16)	−0.61* (0.20)	−0.47* (0.23)	−0.42 (0.26)
Self-control × externalizing behavior influence	−1.20 (0.93)	0.49 (0.61)	1.12 (1.16)	0.50 (1.01)

* *p* < .05

Second, the effects of externalizing behavior, self-control, and control variables were estimated. They estimate the effects of externalizing behavior, self-control, and control variables on selection effects. The main effects of the control variables were generally consistent with prior research. Participants’ selection of friends was significantly associated with similarity in gender, location, and class. Self-control was associated with fewer received friendship nominations (negative self-control alter effect) in one network. In another network, self-control was associated with sending out less friendship nominations (negative self-control ego effect). In two networks participants were likely to select their friends based in a similarity in self-control [positive self-control similarity effect, School 1 Cohort 1 *b* (*SE*) = 0.32 (0.11), School 1 Cohort 1, *b* (*SE*) = 0.93 (0.20)]. In one network the number of friendship nominations participants receive was associated with externalizing behavior (positive externalizing alter effect), and in two networks the number of friendship nominations send out was associated with externalizing behavior (positive externalizing behavior ego effect). In three networks, participants base their friendship on similarity in externalizing behavior [School 1 Cohort *b* (*SE*) = 10.45 (0.19), School 1 Cohort 2 *b* (*SE*) = 0.99 (0.13), School 2 Cohort 2 *b* (*SE*) = 0.86 (0.37)]. Neither of the interaction effects between self-control and selection based on similarity in externalizing behavior reached significance: self-control did not moderate the likelihood to select friends or to be selected as a friend based on similarity in externalizing behavior.

Third, the change in externalizing behavior dynamics was estimated. These behavior dynamics model the change in externalizing behavior. Results revealed a significant negative linear effect in all networks: externalizing behavior increased over time. Furthermore, there was also a positive quadratic effect for externalizing behavior in cohort 1 of School 1 and cohort 1 of School 2. This indicates that externalizing behavior has a tendency to escalate once it develops: participants were likely to either engage in multiple externalizing behaviors or engage in none. In three out of four networks, participants were influenced by their friends; participants adapt their externalizing behavior to become more similar to the average externalizing behavior of their friends [positive externalizing influence effect, School 1 Cohort 1 *b* (*SE*) = 1.15 (0.33), School 1 Cohort 2 *b* (*SE*) = 1.17 (0.27), School 2 Cohort 2 *b* (*SE*) = 1.24 (0.57)]. To test our hypotheses, the role of self-control was estimated in the behavior dynamics. Testing our first hypothesis, in two of the four networks self-control was negatively associated with the development of externalizing behavior [negative effect form self-control, School 1 Cohort 2 *b* (*SE*) = -0.61 (0.20), School 2 Cohort 1 *b* (*SE*) = -0.47 (0.23)]. Thus a lower self-control was associated with a higher likelihood to engage in more externalizing behaviors over time. Testing our second hypothesis, the interaction effect between self-control and externalizing behavior influence was not significant (self-control × total exposure): self-control did not change the likelihood for participants to be influenced by their friends’ externalizing behavior.

## Discussion

This study investigated how self-control affects the co-development of early adolescents’ friendship and externalizing behavior (antisocial behavior, alcohol use, tobacco use). Following after inconsistent findings by previous studies, a more rigorous approach was needed to test the hypotheses that adolescents’ personal level of self-control makes adolescents initiate externalizing behavior regardless of their friends’ externalizing behavior, or if it moderates the extent to which they will initiate externalizing behavior to become more like their friends (e.g., Gardner et al. 2008; McGloin and O’Neill Shermer 2009; Meldrum et al. 2009; Wright et al. 2001). Using stochastic actor-based modelling (SABM; Steglich et al. 2010), a stringent test of these hypothesized moderation effects was possible. The main findings indicate that self-control, in two out of four networks, directly impacts the development of externalizing behavior (in line with hypothesis 1), but that it does not affect if they adapt their friends’ externalizing behavior (not in line with hypothesis 2).

In line with the General Theory of Crime (Gottfredson and Hirschi 1990) and supporting our first hypothesis, self-control rather than the interaction between self-control and friends’ externalizing behavior seems to further the development of externalizing behavior during early adolescence. Self-control is strongly associated with an abundance of negative outcomes for teenagers (Moffitt et al. 2011), which might suggest that adolescents with lower self-control do not need the influence of friends to engage in externalizing behaviors. However, even when taking the direct effects of self-control into consideration, we found that early adolescents do select their friends to match their externalizing behaviors and also adapt their externalizing behavior to become more similar to their friends. Furthermore, the lack of a significant interaction between self-control and friends’ influence indicates that adolescents are influenced by their peers regardless of their self-control level (not supporting our second hypothesis). Thus, having lower self-control and having friends who engage in externalizing behavior are both predictive of engaging in externalizing behavior, and the effect of self-control seems additive rather than synergistic. As there was no interaction between self-control and externalizing behavior selection or influence effects, the selection and influence processes causing similarity on externalizing behavior between early adolescents are similar for those with a low self-control and their peers. However, as early adolescents with a low self-control are in some networks more likely to develop externalizing behaviors they are also likely to befriend peers with similarly high externalizing behaviors and to influence their friends to engage in externalizing behaviors.

In addition to our main research question focusing on self-control and the development of externalizing behavior, several other findings emerged. First, previous research using SABM has focused on one behavior rather than on a broader spread of externalizing behaviors. Such studies have shown that early adolescents select friends based on similar externalizing behaviors, and that they are influenced by their friends to engage in externalizing behaviors. This study shows that early adolescents are also likely to select friends based on the presence or absence of externalizing behavior, and also adopt externalizing behaviors based on their friends’ externalizing behaviors. Furthermore, our study provided a unique test of the association between self-control and friendship and allowed for further testing of some assumptions made by Gottfredson and Hirschi (1990). In line with their expectations, but against our hypothesis based on Young (2011), early adolescents select friends who have similar levels of self-control; the results indicate that adolescents with a lower self-control tend to cluster together (the “birds of a feather” phenomenon). Although the study by Young (2011) took some network characteristics into consideration, the current study was able to control for more network effects (such as reciprocity, and the likelihood to send or receive friendship nominations), and also take friendship selection based on externalizing behaviors into account. In addition, our findings also indicate that low self-control does not (or only in some networks) necessarily reduce the number of friends that early adolescents have. Only in one of the four networks was there a negative effect of self-control on sending friendship nomination and in another network on receiving friendship nominations. Thus, only in some networks early adolescents with low self-control might indeed have difficulty making and keeping friends as they are as likely or less likely to send and receive friendship nominations, and they seem to end up surrounded with friends with a similar level of self-control; in line with the expectations of Gottfredson and Hirschi (1990).

This study has several strengths, such as a focus on early adolescence and early engagement in externalizing behavior, as participants were just entering secondary school, and were on average only around 12.5 years old. This allowed us to study the very beginning of the spread of externalizing behavior during early adolescence. Furthermore, we used a direct measure of externalizing behavior of peers rather than an indirect measure (when adolescents estimate the externalizing behavior of their peers)—a major shortcoming in other studies (see Meldrum et al. 2009). Moreover, we were able to perform our analyses on several similar networks. This allowed to compare replication of robust findings across different networks.

Future studies could build on our findings in several ways. First, by taking other network structures into account, such as cliques (McGloin and O’Neill Shermer 2009), or classroom attitudes towards externalizing behavior (Rambaran et al. 2013). Investigating cliques might be especially important when studying self-control as our results show that the early adolescents tend to select their friends based on the friends’ similar level of self-control, and therefore cliques of early adolescents who have a low self-control and engage in externalizing behavior can be expected (Gottfredson and Hirschi 1990). Second, as we focused on externalizing behavior during the last month before assessments, behaviors which took place longer than a month before the assessment might have been missed. Future studies could investigate if similar results can be found taking the whole development of externalizing behavior between assessments into consideration. However, taking last month prevalence into account does ensure that we have captured the externalizing behaviors of adolescents who tend to be engaged in externalizing behaviors on a more structural basis. Third, our study focused on the occurrence rather than frequency of externalizing behavior as we were interested in the very beginning of spread of adolescent externalizing behavior rather than the further quantitative development of these behaviors. However, this leaves room for investigation on the role of self-control in the development of the frequency of externalizing behavior. Furthermore, instead of focusing on friendship in school, future studies could investigate peers or also include friendships outside of the school context. Moreover, it would be interesting to learn more about changes over time later in adolescence. Future studies could compare these findings with findings in older adolescents.

## Conclusion

This study shows that both personal self-control and friendship are important forces in the development of externalizing behavior. However, our findings show that these two effects co-exist rather than interact. Both having a lower self-control and having friends who engage in externalizing behavior increases adolescents’ chance to increase the number of externalizing behaviors they engage in. Our findings also suggest that self-control, although it is very important in the development of adolescents (Moffitt et al. 2011), is not always associated with the development of externalizing behavior when taking the effects of friendships into account. To prevent the spread of externalizing behavior in young teens, it will be important to focus on adolescents who have friends who engage in externalizing behavior and on adolescents with low self-control. Although increasing self-control might be a good way to prevent externalizing behaviors for several reasons (Piquero et al. 2010), this might not be effective in all friendship networks. It might be beneficial to train self-control already during childhood to prevent adolescents to engage in behaviors which could ensnare them in longer term negative behavior (Moffitt et al. 2011). Furthermore, as self-control seems to be more malleable for females than for males (Piquero et al. 2010), perhaps especially in classes with more females training self-control could be a way to prevent externalizing behaviors. Future studies should further investigate what makes early adolescents more susceptible to peer influence processes on externalizing behavior.
